# Association between Dietary Fatty Acid Patterns and Colorectal Cancer Risk: A Large-Scale Case-Control Study in China

**DOI:** 10.3390/nu14204375

**Published:** 2022-10-19

**Authors:** Kexin Tu, Ting Ma, Ruolin Zhou, Lei Xu, Yujing Fang, Caixia Zhang

**Affiliations:** 1 Department of Epidemiology, School of Public Health, Sun Yat-sen University, Guangzhou 510080, China; 2 Department of Experimental Research, Sun Yat-sen University Cancer Center, State Key Laboratory of Oncology in South China, Collaborative Innovation Center for Cancer Medicine, 651 Dongfeng Road East, Guangzhou 510060, China

**Keywords:** fatty acid patterns, colorectal cancer risk, factor analysis, case-control study

## Abstract

Associations of dietary fatty acids with the risk of colorectal cancer (CRC) remain controversial. The objective of this study was to examine whether dietary-derived fatty acid patterns were related to CRC risk among Chinese people. A total of 2806 CRC patients and 2806 frequency-matched controls were interviewed in this case-control study between July 2010 and May 2021. A food frequency questionnaire was used to gather information on dietary intake. Four fatty acid patterns were identified using factor analysis. The even-long-chain fatty acid pattern had no statistically significant association with CRC risk (adjusted Odds ratio (aOR), 1.16; 95% confidence interval (CI), 0.97–1.39; *p*_trend_ = 0.129). However, significant inverse associations were found between the medium-chain and long-chain saturated fatty acid (SFA) pattern (aOR, 0.34; 95%CI, 0.27–0.42), the highly unsaturated fatty acid pattern (aOR, 0.73; 95%CI, 0.60–0.88), the odd-chain fatty acid pattern (aOR, 0.69; 95%CI, 0.57–0.83), and CRC risk. The interaction between fatty acid patterns and sex was observed, and the association between the highly unsaturated fatty acid pattern and CRC risk differed by subsite. In conclusion, increasing the intakes of foods rich in medium-chain SFAs, highly unsaturated fatty acids, and odd-chain fatty acids may be related to a lower risk of CRC.

## 1. Introduction

Colorectal cancer (CRC) seriously endangers the health of the population worldwide. According to GLOBOCAN 2020 database, there were 1.9 million new cases and 0.9 million deaths of CRC worldwide in 2020, ranking third and second among all cancers [1]. According to Chinese tumor registry data, the number of new CRC cases and deaths in China were 408,000 and 196,000 in 2016, ranking second and fourth among all cancers. The disability-adjusted life year and economic burden due to CRC continues to increase in the Chinese population [2].

Both hereditary and environmental factors contribute to the development of CRC [3]. Based on twin and family studies, estimates for the heritability of CRC range from 12%–35% [4,5]. Modifiable factors for a higher risk of CRC include smoking, drinking, obesity, physical inactivity, and certain dietary factors [6,7,8]. Pakiet et al. noted that the introduction of the Western diet, which is distinguished by large levels of saturated fatty acid (SFA), can be blamed for the recent rise in the incidence of CRC in Eastern populations [9]. Accumulating evidence suggests that a healthy diet can prevent the development of CRC. However, until now, the results of epidemiological studies on specific associations of dietary fatty acids with the risk of CRC remain inconsistent.

According to the number of carbons in their chains, fatty acids were classified as short-chain (<6 carbons), medium-chain (6–12 carbons), or long-chain fatty acids (>12 carbons) [10]. According to the degree of saturation of carbon chains, fatty acids can be divided into SFA and unsaturated fatty acids, the latter including monounsaturated fatty acid (MUFA) and polyunsaturated fatty acid (PUFA) [11]. Highly unsaturated fatty acids contain three or more double bonds and the length of the chain is more than 20 carbon atoms [12]. A meta-analysis [13] and a prospective cohort study [14] found no significant association between dietary total fatty acids, SFA, MUFA, PUFA, *n*-6 PUFAs, *n*-3 PUFAs, and CRC risk. Nevertheless, a case-control study suggested that SFA and MUFA-rich diets were related to an increased risk of CRC [15]. A prospective study found that dietary *n*-3 PUFAs were negatively related to CRC risk, and dietary *n*-6 PUFAs were positively related to rectal cancer risk [16]. One possible explanation for inconsistent findings in epidemiological studies based on single SFA, MUFA, and PUFA intakes is that they do not take into account the possibly varied effects of different fatty acids on the risk of CRC [17]. Additionally, the conflicting results can be explained by the fact that dietary nutrients are often correlated and eating patterns are complex, making it hard to isolate the specific effects of a single nutrient.

One of the approaches to moving the field of diet and cancer research forward is the assessment of the overall, rather than the individual, components of dietary patterns [18]. The fatty acid pattern refers to summarizing all fatty acids into several unrelated “components” or “factors” using a statistical method of dimension reduction. There has been some research on the association between fatty acid patterns and several disorders, including cardiovascular disease [19], prostate cancer [20], breast cancer [21], oral cancer [22], and cognitive function [17]. However, no studies have been reported that apply pattern analysis methods to investigate the association between fatty acids and CRC risk.

This study aimed to identify the main dietary fatty acid patterns of Chinese people and to discover how these patterns were related to CRC risk. We hypothesized that pattern analysis could better capture the interactions of dietary fatty acids and their associations with CRC risk, and a dietary fatty acid pattern high in SFAs would be associated with a higher risk of CRC, while a dietary fatty acid pattern characterized by *n*-3 PUFAs would be associated with a lower risk of CRC.

## 2. Materials and Methods

### 2.1. Study Population

Data were collected from participants in a case-control study that commenced in July 2010. Detailed recruiting procedures and data collection were outlined elsewhere [23].

#### 2.1.1. Inclusion and Exclusion Criteria for Cases

Potential patients were recruited consecutively at the Sun Yat-sen University Cancer Center in Guangzhou, China. Inclusion criteria included patients diagnosed with CRC by histopathology within 3 months preceding the interview, those that were 30–75 years old, and those who were natives of Guangdong province or having lived in Guangdong for at least 5 years.

Exclusion criteria were a history of other cancers, the inability to communicate with others, and an unreasonable daily energy consumption (<600 or >3500 kcal/d for women, <800 or >4200 kcal/d for men).

Between July 2010 and May 2021, 3174 CRC patients met the criteria, and 2833 were ultimately interviewed. The non-response rate was estimated at 10.74%. Finally, 2806 CRC patients were included in the analysis. Of these patients, 1797 were diagnosed with colon cancer and 1009 with rectal cancer.

#### 2.1.2. Inclusion and Exclusion Criteria for Controls

Controls were matched by 5-year age groups and sex, according to frequency. During the enrollment of patients, two control groups were simultaneously recruited. Hospital-derived controls were recruited consecutively at the First Affiliated Hospital, Sun Yat-sen University, and they were required to have no diseases with an apparent association with dietary intake. Specifically, they were recruited in otolaryngology, plastic surgery, and vascular surgery. Another group of controls was recruited from communities in the cases’ cities through community advertisements, flyers, written invitations, or recommendations from research participants. Inclusion criteria were similar to the cases except that eligible controls did not have CRC. Between July 2010 and May 2021, 1743 hospital-derived controls met the criteria and 1504 were ultimately interviewed. The non-response rate was estimated at 13.71%. In addition, 1302 healthy controls were recruited from the community. Totally, the analysis consisted of 2806 controls.

This study was carried out under the ethical standards set out in the 1964 Declaration of Helsinki and its subsequent amendments. The protocol and procedures for this study were endorsed by the Ethics Committee of the School of Public Health, Sun Yat-sen University. An informed consent form was signed by each study subject.

### 2.2. Data Collection

Face-to-face interviews were conducted by trained interviewers using a structured questionnaire to gather data on participants’ sociodemographic characteristics (age, men/women, marital status, residence, education, occupation, income), height and weight, lifestyles (physical activity, smoking and drinking status, passive smoking), and history of cancer in first-degree relatives. Women’s menstrual history was also collected. Body mass index (BMI) is a person’s weight in kilograms divided by the square of height in meters. Physical activity included occupational physical activity and household and recreational physical activity, which were assessed on the basis of self-reported physical activities during the past year. Investigations into the physical activity of occupational subjects resulted in the classifications of sedentary, light, moderate, and heavy activity occupations, with examples provided. Metabolic equivalent task (MET)-hours/week was used to measure the household and recreational physical activity. We obtained the mean MET-hour score of activities according to the Compendium of Physical Activities [24,25,26], and MET-hours/week of the activity was calculated by multiplying the mean MET-hour score by the time spent. In the analysis, ever smokers contained regular smokers and former smokers. Regular smokers are those who smoke one or more cigarettes daily for more than 6 continuous months. Former smokers are those who were classified as regular smokers but have not smoked in the past 6 months. The definition of passive smoking is being exposed to another person’s tobacco smoke for a minimum of 5 min each day over the past 5 years. Regular drinkers are those who consumed alcohol at least one time weekly over the past 12 months.

### 2.3. Dietary Assessments

A validated food frequency questionnaire (FFQ) was used to collect dietary intakes. FFQ included 81 food and beverage items, including 12 types of cereals, 7 types of legumes, 18 types of vegetables, 11 types of fruits, 18 types of meat, 2 types of eggs, 8 types of dairy products, 3 types of beverages and soups, and 2 types of mushrooms and nuts. There was a total of 244 specific foods. Intake frequency and portion size in the preceding 12 months prior to diagnosis for cases or interviews for controls were asked to report. The validity and reproducibility of the FFQ were validated elsewhere [27] and FFQ was used in several previous studies [28,29,30]. Comparing the FFQ and the six three-day dietary records, the energy-adjusted Pearson correlation coefficients were 0.30–0.68 for food groups, 0.25–0.65 for nutrients, 0.34 for total fat, 0.37 for SFA, and 0.32 for unsaturated fatty acids. For the FFQ reproducibility, the correlation coefficients for total fat, SFA and MUFA, and PUFA were 0.61, 0.63, and 0.58, respectively [23].

The intakes of energy and nutrients were computed based on the China Food Composition Table 2002 [31], China Food Composition Tables Standard Edition [32], and the 2019 Taiwan food nutrition composition database. The China Food Composition Table provides the fatty acid profiles of each 100 g of food. If the food items in FFQ contain more than one specific food, then the fatty acid profile is the average value of those foods. This analysis contained 32 types of fatty acids, including 14 types of SFAs, 7 types of MUFAs, and 11 types of PUFAs. SFAs contain caproic acid (6:0), caprylic acid (8:0), capric acid (10:0), undecanoic acid (11:0), lauric acid (12:0), tridecanoic acid (13:0), myristic acid (14:0), pentadecanoic acid (15:0), palmitic acid (16:0), heptadecanoic acid (17:0), stearic acid (18:0), nonadecanoic acid (19:0), arachidic acid (20:0), and behenic acid (22:0). MUFAs contain myristoleic acid (14:1), pentadecenoic acid (15:1), palmitoleic acid (16:1), heptadecenoic acid (17:1), oleic acid (18:1), gadoleic acid (20:1), and erucic acid (22:1). PUFAs contain hexadecadienoic acid (16:2), linoleic acid (LA, 18:2), alpha-linolenic acid (ALA, 18:3), eicosadienoic acid (20:2), eicosatrienoic acid (20:3), arachidonic acid (AA, 20:4), eicosapentaenoic acid (EPA, 20:5), docosatrienoic acid (22:3), docosatetraenoic acid (22:4), docosapentaenoic acid (DPA, 22:5), and docosahexaenoic acid (DHA, 22:6). *N*-3 long-chain polyunsaturated fatty acids (*n*-3 LC-PUFAs) contains EPA, DPA, and DHA.

### 2.4. Factor Analysis of Fatty Acid Pattern

Factor analysis was conducted to derive fatty acid patterns based on 32 individual dietary fatty acid levels of the controls. Firstly, the Kaiser-Meyer-Olkin (KMO) test was performed on 32 fatty acids, and the factor analysis was determined according to the KMO test statistic. Principal component analysis (PCA) with varimax rotation was used to identify dietary fatty acid patterns, resulting in uncorrelated patterns and a simpler and more understandable structure. Next, the scree plot, the percentage of variance explained, and the interpretability of the found components were used to determine the number of factors to extract. The factor loading of each fatty acid was used to measure the contribution to patterns, and an absolute value greater than 0.5 was considered to be a significant contributor. Patterns were named based on the major contributing fatty acids. For each participant, factor scores for the first four factors were calculated from the rotated loading factors and were modeled using logistic regression. Factor scores of each pattern represent the degree of coincidence between the study subjects’ dietary fatty acid intakes and the pattern. Higher factor scores indicate a greater coincidence.

### 2.5. Statistical Analysis

The analysis was conducted with IBM SPSS Statistics for Windows, Version 26.0. Armonk, NY, USA: IBM Corp. The daily intakes of foods and fatty acids were logarithmically transformed, and a residual method was used to adjust the energy [33]. To compare the demographic and dietary intake variables for cases and controls, the Wilcoxon rank-sum test was used for continuous variables, and the chi-square test was used for categorical variables. For each fatty acid pattern, the factor scores of the control group were divided into quintiles by sex. Based on the factor score values corresponding to the quintiles, all study subjects were classified into five classes (quintile1 (Q1)–quintile5 (Q5)). Q1 indicates that the fatty acid intakes did not conform to the pattern, and Q5 indicates that the fatty acid intakes conformed highly to the pattern. In the multivariable unconditional logistic regression models, odds ratios (ORs) and 95% confidence intervals (CIs) were calculated for Q2–Q5, respectively, using Q1 as the reference. The value of *p* for trend was calculated by placing Q1–Q5 as a continuous variable in the regression models. Based on previous studies and the comparisons of case and control characteristics, the following potential confounding variables were identified: age (years), marital status, residence, education, occupation, income, occupational activity, MET, BMI, smoking and drinking status, history of cancer in first-degree relatives, and the daily intakes of energy, vegetables, and fruits.

To determine whether the association between dietary fatty acid patterns and CRC risk was modified by sex, a sex-stratified analysis was performed. The value of *p*_interaction_ was calculated by placing the multiplication terms of sex and fatty acid pattern into the regression models. A subgroup analysis was conducted based on the cancer site (colon or rectum) of CRC patients, and the value of *p*_heterogeneity_ was based on cases only. In all analyses, *p* values were two-sided, and the differences were determined to be statistically significant at the *p* < 0.05 level.

## 3. Results

### 3.1. General Characteristics

Table 1 displays the characteristics and dietary intakes of fatty acids among CRC cases and controls. The average (SD) age of the 2806 CRC cases was 57.11 (10.28) years, with 1606 of the cases being men (57.23%). Compared to controls, more cases were married, lived in rural areas, had a poor level of education, and were blue-collar workers, farmer/others. Correspondingly, the cases performed a higher intensity of occupational physical activities and were less likely to engage in household and leisure-time activities. In addition, the cases had a lower mean BMI, a higher proportion of ever smokers and regular drinkers, and a higher proportion of history of cancer in first-degree relatives. Cases had a lower daily intake of energy, vegetables, fruits, and PUFA, but not MUFA.

### 3.2. Dietary Fatty Acid Patterns

**Figure 1:**
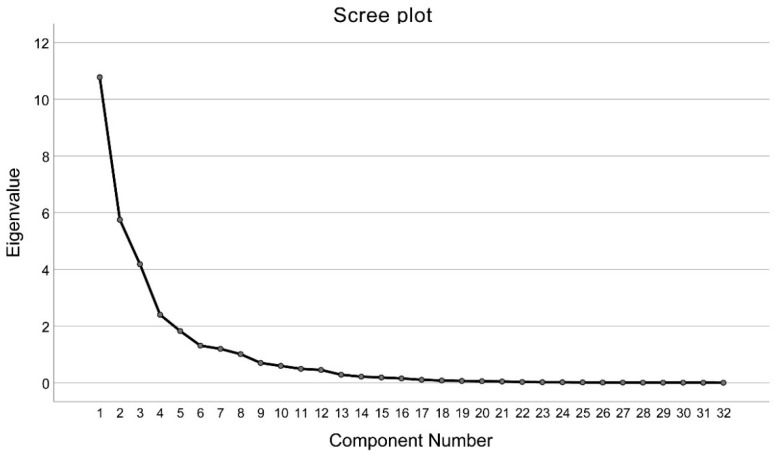
The scree plot of factor analysis by using dietary fatty acids data among 2806 controls.

Factor analysis showed that the KMO test statistic was 0.725 > 0.5, demonstrating a high and appropriate correlation between the fatty acids. Four factors were finally retained, and the scree plot is shown in Figure 1. Together, 72.21% of the variance in the data from the controls was explained by four factors, with the corresponding contributions from factors 1–4 being 24.16%, 20.46%, 14.42%, and 13.17%.

Table 2 displays factor loadings for 32 fatty acids. Factor 1 was named the even-long-chain fatty acid pattern for containing 16-, 18-, and 20-carbon fatty acids, with high factor loading for oleic acid (18:1), palmitic acid (16:0), stearic acid (18:0), palmitoleic acid (16:1), arachidic acid (20:0), hexadecadienoic acid (16:2), ALA, eicosadienoic acid (20:2), LA and gadoleic acid (20:1). Factor 2 was characterized as the medium-chain and long-chain saturated fatty acid (SFA) pattern, with high factor loading for capric acid (10:0), caproic acid (6:0), myristoleic acid (14:1), tridecanoic acid (13:0), myristic acid (14:0), eicosatrienoic acid (20:3), caprylic acid (8:0), and lauric acid (12:0). Factor 3 was named the highly unsaturated fatty acid pattern for loading primarily and positively onto EPA, docosatetraenoic acid (22:4), DHA, DPA, docosatrienoic acid (22:3), and AA. Factor 4, the odd-chain fatty acid pattern, was characterized by higher levels of heptadecenoic acid (17:1), pentadecenoic acid (15:1), heptadecanoic acid (17:0), undecanoic acid (11:0), and pentadecanoic acid (15:0).

### 3.3. Dietary Fatty Acid Patterns and CRC Risk

After adjusting for various confounding variables, the even-long-chain fatty acid pattern was discovered to be related to higher CRC risk, although it was not statistically significant (adjusted OR_Q5 vs. Q1_ (aOR), 1.16; 95%CI, 0.97–1.39; *p*_trend_ = 0.129). On the other hand, inverse associations were found between the medium-chain and long-chain SFA pattern (aOR, 0.34; 95%CI, 0.27–0.42; *p*_trend_ < 0.001), the highly unsaturated fatty acid pattern (aOR, 0.73; 95%CI, 0.60–0.88; *p*_trend_ < 0.001), the odd-chain fatty acid pattern (aOR, 0.69; 95%CI, 0.57–0.83; *p*_trend_ < 0.001), and the risk of CRC (Table 3).

**Figure 2:**
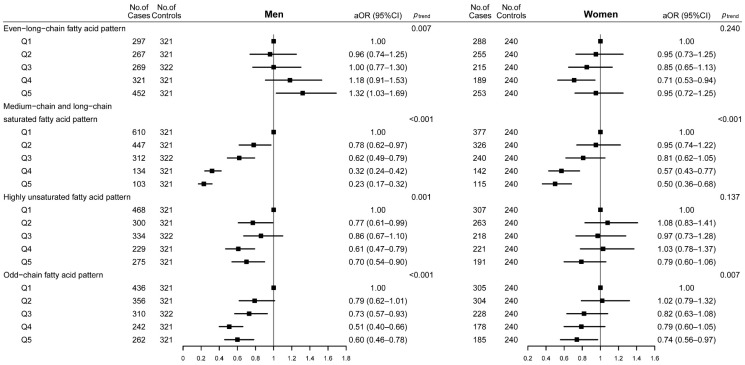
Adjusted odds ratios and 95% confidence intervals of colorectal cancer across quintiles of dietary fatty acid patterns according to sex. Abbreviations: Q, quintile; Q1–Q5 means the lowest quintile to the highest quintile. aOR, adjusted odds ratio; CI, confidence interval. Adjusted confounding variables included age (years), marital status, residence, education, occupation, income, occupational activity, MET, BMI, smoking and drinking status, history of cancer in first-degree relatives, and daily intakes of energy, vegetables, and fruits. The value of *p* for trend was calculated by placing Q1–Q5 as a continuous variable in the regression models.

Sex-stratified analyses showed that the even-long-chain fatty acid pattern was associated with an increased CRC risk only in men but not in women (*p*_interaction_ = 0.009). The medium-chain and long-chain SFA pattern was associated with a decreased CRC risk in both men and women, but it was stronger in men (*p*_interaction_ = 0.006). The odd-chain fatty acid pattern was significantly related to lower CRC risk in both men and women. For the highly unsaturated fatty acid pattern, a significant negative association was found only in men. However, the interactions between these two above-mentioned patterns and sex with CRC risk were not significant (*p*_interaction_ = 0.732 for the odd-chain fatty acid pattern, *p*_interaction_ = 0.577 for the highly unsaturated fatty acid pattern, respectively) (Figure 2) (the values of *p*_interaction_ were not shown in the figure).

**Figure 3:**
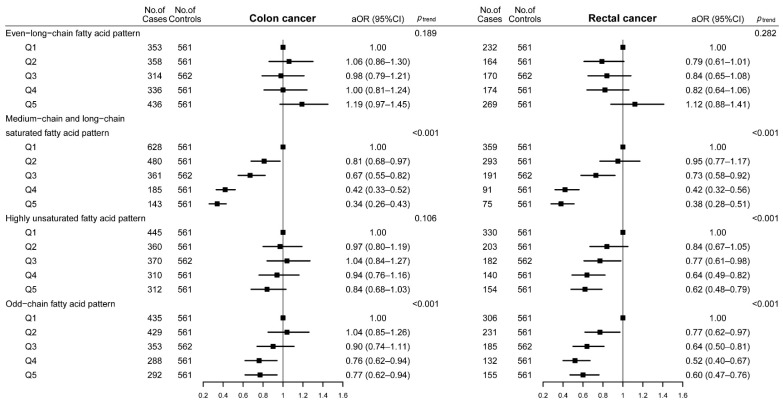
Adjusted odds ratios and 95% confidence intervals of colorectal cancer across quintiles of dietary fatty acid patterns according to cancer site. Abbreviations: Q, quintile; Q1–Q5 means the lowest quintile to the highest quintile. aOR, adjusted odds ratio; CI, confidence interval. Adjusted confounding variables included age (years), marital status, residence, education, occupation, income, occupational activity, MET, BMI, smoking and drinking status, history of cancer in first-degree relatives, and daily intakes of energy, vegetables, and fruits. The value of *p* for trend was calculated by placing Q1–Q5 as a continuous variable in the regression models.

The results of subgroup analyses by cancer site revealed that the medium-chain and long-chain SFA pattern and the odd-chain fatty acid pattern were associated with a decreased CRC risk in both colon and rectal cancer. The even-long-chain fatty acid pattern was not associated with the risk of colon or rectal cancer. However, the highly unsaturated fatty acid pattern was found to be significantly associated with a decreased rectal cancer risk only (aOR, 0.62; 95%CI, 0.48–0.79; *p*_trend_ < 0.001; *p*_heterogeneity_ = 0.001) (Figure 3) (the values of *p*_heterogeneity_ were not shown in the figure).

## 4. Discussion

The objective of this study was to examine whether dietary-derived fatty acid patterns were related to CRC risk among Chinese people. We identified four dietary fatty acid patterns using PCA. No significant association was found between the even-long-chain fatty acid pattern and the risk of CRC. However, the medium-chain and long-chain SFA pattern, the highly unsaturated fatty acid pattern, and the odd-chain fatty acid pattern were observed to be inversely associated with CRC risk.

The even-long-chain fatty acid pattern (Factor 1) contains the main fatty acids used in the human body, especially 16- and 18-carbon fatty acids. No significant association was found with the risk of CRC in our study. One possible explanation is that this pattern contains a variety of fatty acids, which have different associations with CRC risk. Two prospective studies from EPIC [34,35] and a Mendelian randomization analysis [36] observed a positive association between erythrocyte or plasma stearic acid (18:0) and CRC risk. There was also a positive association between plasma phospholipids [16] or erythrocyte membranes [37] palmitic acid (16:0) and CRC risk. However, the effect of oleic acid (18:1) and palmitoleic acid (16:1) on CRC risk is unclear. A Mendelian randomization analysis found that plasma oleic acid (18:1) and palmitoleic acid (16:1) were inversely associated with CRC risk [36], while a case-control study showed that dietary oleic acid (18:1) was not associated with colon cancer risk [38]. An EPIC study suggested that dietary palmitoleic acid (16:1), originating from margarine, fried fats, and meat products, was associated with increased CRC risk [35].

ALA is known to play the crucial metabolic function of being a precursor to EPA and DHA [39]. However, a review concluded that the rate of transformation of ALA into DHA is almost nil among adults [40]. Meta-analyses showed that dietary ALA and LA were not associated with CRC risk [14,41,42]. However, an inverse association with CRC or colon cancer risk was found for blood ALA [42] and plasma LA [36,43]. No epidemiological study was reported on the association between hexadecadienoic acid (16:2), arachidic acid (20:0), gadoleic acid (20:1), eicosadienoic acid (20:2), and CRC risk.

Contrary to our hypothesis, the medium-chain and long-chain SFA pattern (Factor 2) was found to be inversely associated with CRC risk. This pattern contains 6–14-carbon SFA except for undecanoic acid (11:0), as well as myristoleic acid (14:1) and eicosatrienoic acid (20:3). The main dietary sources of these fatty acids are ice cream, milk and milk powder, and cheeses among Chinese people’s daily diet. A cellular study [44] and an in silico and in vitro study [45] found anti-cancer effects of caproic acid (6:0), caprylic acid (8:0), capric acid (10:0), and lauric acid (12:0). Myristoleic acid (14:1) and eicosatrienoic acid (20:3) are rare in nature. A study [46] suggested that myristoleic acid (14:1) is a cytotoxic component and can induce apoptosis and necrosis in human prostatic LNCaP cells. A review indicated that eicosatrienoic acid (20:3) has anti-inflammatory properties [47].

So far, few epidemiological studies have examined the relationship between these fatty acids and CRC risk. A black Women’s Health Study found no association between dietary caproic acid (6:0), caprylic acid (8:0), capric acid (10:0), and lauric acid (12:0) and CRC risk [48]. A prospective study [35], but not others [16,34], found an inverse association between dietary or plasma myristic acid (14:0) and CRC risk. A cohort study derived a short and medium-chain SFA pattern using PCA and found that this pattern was associated with greater global cognitive function [17]. Our observation of the inverse association of the medium-chain and long-chain SFA pattern with CRC risk probably indicates the different effects of various types of SFAs and reveals the potential health effects of medium-chain SFAs on CRC risk. Future studies could pay more attention to the effects of SFAs with different carbon atomic numbers on CRC.

The highly unsaturated fatty acid pattern (Factor 3) characterized by *n*-3 LC-PUFAs and *n*-6 PUFAs was found to be inversely associated with CRC risk in our study, which was consistent with our hypothesis. One previous study also derived a pattern characterized by *n*-3 LC-PUFAs and AA, but no association was found with cognitive function [17]. A meta-analysis [49] and a study from EPIC [50] found that dietary *n*-3 LC-PUFAs, EPA, DPA, and DHA were inversely associated with CRC risk. The main dietary source of *n*-3 LC-PUFAs is fish, especially oily fish, which are inversely associated with CRC risk [50]. So far, there is no consensus on the association between *n*-6 PUFAs and CRC risk. It was previously believed that dietary or plasma *n*-6 PUFAs were not associated with [41,51,52] or positively associated with CRC risk, especially AA [36,53,54]. However, some studies indicated that *n*-6 PUFAs may be engaged in the anti-cancer process [55,56,57,58]. One study in the United States found that dietary *n*-6 PUFAs were inversely related to CRC risk in men [59]. Further research is necessary to identify the role of *n*-6 PUFAs.

Our study suggested that the odd-chain fatty acid pattern (Factor 4) was inversely associated with CRC risk. The physiological significance of odd-chain SFAs was generally considered negligible. However, recent studies have shown that pentadecanoic acid (15:0) and heptadecanoic acid (17:0) are beneficial to health and may reduce the risk of many diseases [60,61,62,63]. Two studies found an inverse association between dietary pentadecanoic acid (15:0), red blood cell heptadecanoic acid (17:0), and CRC risk [16,34]. Higher World Cancer Research Fund/American Institute for Cancer Research (WCRF/AICR) scores were characterized by metabolic signatures of increased pentadecanoic acid (15:0) and heptadecanoic acid (17:0). Higher WCRF/AICR scores were reported to be associated with a lower risk of CRC [64]. There are few studies on the role of pentadecenoic acid (15:1) and heptadecenoic acid (17:1) in human health. A cellular study found that heptadecenoic acid (17:1) in the Ganoderma spores has a slight suppressive function on the proliferation of human cancer cells [65]. Odd-chain fatty acids are largely derived through de novo synthesis in rumen bacteria and the mammary gland of ruminants [66]. Therefore, odd-chain fatty acids can be obtained mainly from ruminant milk.

The results of this study suggested that the association between dietary fatty acid patterns and CRC risk could be modified by sex. The even-long-chain fatty acid pattern exhibited a significant positive association with CRC risk only in men. The inverse association of the medium-chain and long-chain SFA pattern with CRC risk was stronger in men than women. Some previous studies observed a sex-specific difference in *n*-6 PUFAs, *n*-3 PUFAs, and marine *n*-3 PUFAs [59,67]. It is challenging to interpret the sex-specific difference due to the limited evidence. One possible explanation is that estrogen may change the metabolism of fatty acids by altering the usage and oxidation of fatty acids [51]. An in vitro study found the reduction of palmitic acid (16:0) and the increase of palmitoleic acid (16:1) and LA in estradiol-treated human CRC stem cells [68]. Molecular biology studies have also identified a possible protective effect of estrogen against CRC, but the mechanism is unclear [69].

In our study, the association between the highly unsaturated fatty acid pattern with CRC risk differed by subsite. This pattern was inversely related to rectal cancer but not to colon cancer. No heterogeneity by cancer site was found for the other three fatty acid patterns. It is difficult to interpret the subsite heterogeneity found in our study in terms of biological plausibility. Molecular features of proximal colon cancer are different when compared with distal colon cancer and rectal cancer [70]. The prospective research from the Melbourne Collaborative Cohort showed that dietary palmitic acid (16:0), stearic acid (18:0), palmitoleic acid (16:1), oleic acid (18:1), and MUFAs were significantly related to higher rectal cancer risk and were not significantly related to a lower risk of colon cancer, and subsite heterogeneity was found (*p*_heterogeneity_ < 0.05) [16]. In another prospective study, dietary DPA, DHA, and *n*-3 LC-PUFAs were significantly associated with lower colon cancer risk among women, and they were not significantly associated with a higher risk of rectal cancer; the result of the heterogeneity test was not reported [52].

One of this study’s strengths is that it is the first study to examine dietary fatty acid patterns in relation to CRC risk. By converting the data from a group of strongly correlated fatty acids into unrelated factors, the PCA method enabled us to assess the impact of all fatty acids simultaneously. Additionally, the sample size of this study was large enough to provide sufficient statistical validity for the detection of a convincing association. Notably, in addition to poor dietary habits, other risk factors such as obesity, smoking, alcohol consumption, sedentary behavior, and exposure to pesticides are also associated with an increased risk of CRC [71,72,73,74,75]. We excluded the effect of these risk factors mainly by adjusting them as confounding factors in the multivariable models. In addition, our study revealed that dietary fatty acids might have the potential to serve as biomarkers of a complex diet due to the heterogeneous variation in diet across different regions of China.

The limitations of our study should be taken into account. Firstly, CRC patients were recruited from only one hospital, which might lead to selection bias. However, Sun Yat-sen University Cancer Center is the biggest cancer center in the South China region. Studies have shown that the clinical traits of CRC patients recruited from this cancer center were comparable to CRC patients from other large hospitals in Guangdong Province [76] or across the country [77]. Secondly, information bias existed in this study as well. The non-differential misclassification might weaken the actual association between dietary fatty acid patterns and CRC risk. To minimize the recall bias of study subjects in the investigation, we recruited incident CRC cases who were interviewed no more than 3 months after diagnosis. Thirdly, due to the lack of data on trans fatty acids in the China Food Composition Table, we did not include them in our analyses. Fourthly, some of the decisions made in the PCA method are subjective, such as determining the number of factors to be extracted, the usage of rotation methods, and the naming of the various factors. The results may be different when using different orthogonal or oblique rotation methods. Finally, although animal experiments have found that high-fat diets could alter the composition of the intestinal microbiota [78,79], which was related to an increased incidence of tumors [80,81], our study did not examine how various fatty acids alter the human gut microbial profile and influence the risk of CRC. Further studies are needed to prospectively investigate this issue.

## 5. Conclusions

This study showed that dietary fatty acid patterns may be associated with CRC risk, which have not been identified in previous relevant studies. The dietary medium-chain and long-chain SFA pattern, the highly unsaturated fatty acid pattern, and the odd-chain fatty acid pattern were inversely associated with CRC risk. Ice cream, milk, milk powder, and cheeses are the major dietary sources of the medium-chain and long-chain SFA pattern in the Chinese people’s daily diet. The major dietary sources of the highly unsaturated fatty acid pattern and the odd-chain fatty acid pattern are oily fish and ruminant milk, respectively. The present study indicated that the intakes of these foods mentioned above may be beneficial for CRC prevention. CRC remains a heavy burden in the Chinese population, and dietary intake, as an important and modifiable risk factor, plays a crucial role in the primary prevention of CRC. Our findings provided a theoretical basis for the dietary prevention of CRC in the Chinese population.

## Figures and Tables

**Table 1:** Characteristics and dietary intakes in colorectal cancer cases and controls.

Characteristics/Dietary Intakes	Cases (*n* = 2806)	Controls (*n* = 2806)	*p* ^a^
Age (years), mean (SD)	57.11 (10.28)	57.06 (9.90)	0.747
Men, *n* (%)	1606 (57.23)	1606 (57.23)	>0.99
Married, *n* (%)	2667 (95.05)	2551 (90.91)	<0.001
Rural, *n* (%)	1001 (35.67)	632 (22.52)	<0.001
Education, *n* (%)			<0.001
Unknown	1 (0.04)	3 (0.11)	
Primary school or below	871 (31.04)	631 (22.49)	
Middle school	784 (27.94)	704 (25.09)	
High school/technical school	684 (24.38)	754 (26.87)	
College or above	466 (16.61)	714 (25.45)	
Occupation, *n* (%)			0.004
Administrator/other white-collar	398 (14.18)	488 (17.39)	
Blue-collar worker	624 (22.24)	607 (21.63)	
Farmer/other	1784 (63.58)	1711 (60.98)	
Income (Yuan/month), *n* (%)			<0.001
<2000	381 (13.58)	359 (12.79)	
2001–5000	938 (33.43)	1086 (38.70)	
5001–8000	831 (29.62)	855 (30.47)	
>8001	656 (23.38)	506 (18.03)	
Postmenopausal ^b^	869 (72.42)	885 (73.75)	0.462
Menarche age (years), mean (SD) ^b^	14.81 (2.56)	14.61 (3.03)	0.910
Occupational activity, *n* (%)			<0.001
Nonworking	334 (11.90)	960 (34.21)	
Sedentary	800 (28.51)	580 (20.67)	
Light	774 (27.58)	666 (23.73)	
Moderate	420 (14.97)	268 (9.55)	
Heavy	478 (17.03)	332 (11.83)	
MET (h/week), median (IQR)	27.66 (8.50–52.50)	34.31 (15.75–56.00)	<0.001
BMI (kg/m^2^), mean (SD)	23.34 (3.29)	23.60 (3.13)	0.002
Ever smokers, *n* (%)	1106 (39.42)	857 (30.54)	<0.001
Passive smoking, *n* (%)	795 (28.33)	812 (28.94)	0.616
Regular drinkers, *n* (%)	506 (18.03)	395 (14.08)	<0.001
History of cancer in first-degree relatives, *n* (%)	416 (14.83)	235 (8.37)	<0.001
Dietary intakes, median (IQR) ^c^			
Energy (kcal/d)	1584.66 (1294.21–1940.52)	1646.06 (1366.02–2018.51)	<0.001
Vegetables (g/d)	384.13 (281.16–511.12)	410.75 (304.95–527.37)	<0.001
Fruits (g/d)	86.31 (42.69–144.88)	121.92 (67.60–187.00)	<0.001
Total fat (g/d)	30.42 (22.53–39.23)	30.42 (23.82–37.55)	0.909
SFA (g/d)	11.59 (8.38–15.24)	11.61 (8.94–14.76)	0.532
MUFA (g/d)	13.55 (9.84–17.98)	13.11 (10.13–16.65)	0.009
PUFA (g/d)	4.65 (3.67–5.83)	4.93 (3.93–6.08)	<0.001
*n*-3 LC-PUFAs (mg/d) ^d^	38.29 (18.89–76.04)	44.40 (23.95–79.77)	<0.001

Abbreviations: SD, standard deviation; MET, metabolic equivalent task; IQR, interquartile range; BMI, body mass index; SFA, saturated fatty acid; MUFA, monounsaturated fatty acid; PUFA, polyunsaturated fatty acid; *n*-3 LC-PUFAs, *n*-3 long-chain polyunsaturated fatty acids. ^a^ The Wilcoxon rank-sum test was used for the comparison of continuous variables, and the chi-square test was used for the comparison of categorical variables. ^b^ Only for women. ^c^ A residual method was used to adjust the energy for the daily intake of foods and fatty acids. The average energy consumption among men and women was 1849 kcal/d and 1487 kcal/d. ^d^ Consists of eicosapentaenoic acid (EPA, 20:5), docosapentaenoic acid (DPA, 22:5), and docosahexaenoic acid (DHA, 22:6).

**Table 2:** Factor loadings of four dietary fatty acid patterns determined in 2806 control subjects.

Fatty Acid	Common Name	Factor 1	Factor 2	Factor 3	Factor 4
		Even-Long-Chain Fatty Acid Pattern	Medium-Chain and Long-Chain Saturated Fatty Acid Pattern	Highly Unsaturated Fatty Acid Pattern	Odd-Chain Fatty Acid Pattern
C18:1	oleic	0.962 *	0.053	0.032	0.096
C16:0	palmitic	0.929 *	0.185	0.041	0.152
C18:0	stearic	0.921 *	0.090	−0.017	0.146
C16:1	palmitoleic	0.901 *	0.017	0.161	0.169
C20:0	arachidic	0.874 *	0.161	0.145	0.052
C16:2	hexadecadienoic	0.857 *	−0.100	−0.117	0.088
C18:3	ALA	0.787 *	0.041	0.262	−0.041
C20:2	eicosadienoic	0.774 *	−0.129	−0.167	0.348
C18:2	LA	0.593 *	0.236	0.216	−0.003
C20:1	gadoleic	0.531 *	0.116	0.222	0.136
C10:0	capric	0.025	0.968 *	−0.053	0.116
C6:0	caproic	−0.012	0.930 *	−0.053	0.115
C14:1	myristoleic	0.023	0.912 *	0.048	0.178
C13:0	tridecanoic	0.020	0.840 *	−0.025	0.379
C14:0	myristic	0.405	0.797 *	0.012	0.291
C20:3	eicosatrienoic	0.003	0.785 *	0.284	0.129
C8:0	caprylic	0.033	0.759 *	−0.061	−0.017
C12:0	lauric	0.173	0.658 *	−0.093	0.016
C20:5	EPA	0.074	0.000	0.928 *	0.121
C22:4	docosatetraenoic	0.068	0.055	0.866 *	0.127
C22:6	DHA	0.081	−0.054	0.844 *	0.116
C22:5	DPA	0.127	−0.045	0.843 *	0.098
C22:3	docosatrienoic	0.065	−0.015	0.714 *	−0.031
C20:4	AA	0.540 *	0.004	0.558 *	0.535 *
C17:1	heptadecenoic	0.103	0.282	0.153	0.905 *
C15:1	pentadecenoic	0.063	0.170	0.311	0.852 *
C17:0	heptadecanoic	0.504 *	0.155	−0.054	0.800 *
C11:0	undecanoic	0.093	0.105	0.052	0.763 *
C15:0	pentadecanoic	0.161	0.619 *	0.101	0.662 *
C19:0	nonadecanoic	0.192	0.238	0.412	0.365
C22:0	behenic	0.228	0.425	0.303	0.011
C22:1	erucic	0.120	0.051	0.072	0.001

Abbreviations: ALA, alpha-linolenic acid; LA, linoleic acid; EPA, eicosapentaenoic acid; DHA, docosahexaenoic acid; DPA, docosapentaenoic acid; AA, arachidonic acid. * An absolute value greater than 0.5. The bold fatty acids indicate that they have a major contribution to the pattern.

**Table 3:** Odds ratios and 95% confidence intervals of colorectal cancer across quintiles of dietary fatty acid patterns.

Dietary Fatty Acid Patterns	Q1	Q2	Q3	Q4	Q5	*p*_trend_^b^
Even-long-chain fatty acid pattern						
No. of cases/controls	585/561	522/561	484/562	510/561	705/561	
cOR (95%CI)	1.00	0.89 (0.76–1.05)	0.83 (0.70–0.98)	0.87 (0.74–1.03)	1.21 (1.03–1.42)	0.036
aOR (95%CI) ^a^	1.00	0.94 (0.79–1.13)	0.92 (0.76–1.11)	0.93 (0.77–1.12)	1.16 (0.97–1.39)	0.129
Medium-chain and long-chain saturated fatty acid pattern						
No. of cases/controls	987/561	773/561	552/562	276/561	218/561	
cOR (95%CI)	1.00	0.78 (0.67–0.91)	0.56 (0.48–0.65)	0.28 (0.23–0.33)	0.22 (0.18–0.27)	<0.001
aOR (95%CI) ^a^	1.00	0.85 (0.72–1.00)	0.67 (0.57–0.80)	0.41 (0.33–0.50)	0.34 (0.27–0.42)	<0.001
Highly unsaturated fatty acid pattern						
No. of cases/controls	775/561	563/561	552/562	450/561	466/561	
cOR (95%CI)	1.00	0.73 (0.62–0.85)	0.71 (0.61–0.83)	0.58 (0.49–0.69)	0.60 (0.51–0.71)	<0.001
aOR (95%CI) ^a^	1.00	0.89 (0.75–1.07)	0.89 (0.75–1.07)	0.79 (0.65–0.95)	0.73 (0.60–0.88)	<0.001
Odd-chain fatty acid pattern						
No. of cases/controls	741/561	660/561	538/562	420/561	447/561	
cOR (95%CI)	1.00	0.89 (0.76–1.04)	0.73 (0.62–0.85)	0.57 (0.48–0.67)	0.60 (0.51–0.71)	<0.001
aOR (95%CI) ^a^	1.00	0.92 (0.77–1.09)	0.78 (0.66–0.94)	0.66 (0.55–0.79)	0.69 (0.57–0.83)	<0.001

Abbreviations: Q, quintile; Q1–Q5 means the lowest quintile to the highest quintile. cOR, crude odds ratio; aOR, adjusted odds ratio; CI, confidence interval. ^a^ Adjusted confounding variables included age (years), marital status, residence, education, occupation, income, occupational activity, MET, BMI, smoking and drinking status, history of cancer in first-degree relatives, and daily intakes of energy, vegetables, and fruits. ^b^ The value of *p* for trend was calculated by placing Q1–Q5 as a continuous variable in the regression models.

## Data Availability

The data that support the findings of our study are available from the corresponding author upon reasonable request.
